# The Extracellular Matrix in Bronchopulmonary Dysplasia: Target and Source

**DOI:** 10.3389/fmed.2015.00091

**Published:** 2015-12-23

**Authors:** Ivana Mižíková, Rory E. Morty

**Affiliations:** ^1^Department of Lung Development and Remodelling, Max Planck Institute for Heart and Lung Research, Bad Nauheim, Germany; ^2^Pulmonology, Department of Internal Medicine, University of Giessen and Marburg Lung Center, Giessen, Germany

**Keywords:** bronchopulmonary dysplasia, extracellular matrix, hyperoxia, mechanical ventilation, collagen, elastin, lung development

## Abstract

Bronchopulmonary dysplasia (BPD) is a common complication of preterm birth that contributes significantly to morbidity and mortality in neonatal intensive care units. BPD results from life-saving interventions, such as mechanical ventilation and oxygen supplementation used to manage preterm infants with acute respiratory failure, which may be complicated by pulmonary infection. The pathogenic pathways driving BPD are not well-delineated but include disturbances to the coordinated action of gene expression, cell–cell communication, physical forces, and cell interactions with the extracellular matrix (ECM), which together guide normal lung development. Efforts to further delineate these pathways have been assisted by the use of animal models of BPD, which rely on infection, injurious mechanical ventilation, or oxygen supplementation, where histopathological features of BPD can be mimicked. Notable among these are perturbations to ECM structures, namely, the organization of the elastin and collagen networks in the developing lung. Dysregulated collagen deposition and disturbed elastin fiber organization are pathological hallmarks of clinical and experimental BPD. Strides have been made in understanding the disturbances to ECM production in the developing lung, but much still remains to be discovered about how ECM maturation and turnover are dysregulated in aberrantly developing lungs. This review aims to inform the reader about the state-of-the-art concerning the ECM in BPD, to highlight the gaps in our knowledge and current controversies, and to suggest directions for future work in this exciting and complex area of lung development (patho)biology.

## Bronchopulmonary Dysplasia in Context

**Figure 1:**
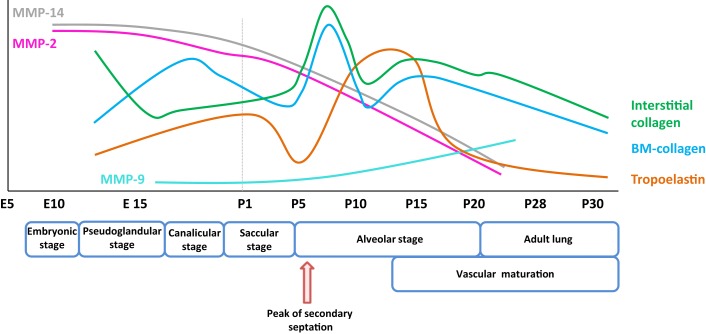
**Expression pattern of selected extracellular matrix components and remodeling enzymes over the course of early and late lung development in mice**. The trends illustrated represent a synthesis of the data presented in several publications (8, 9), and span the embryonic and post-natal lung maturation period. Abbreviations: BM, basement membrane; E, embryonic day; MMP, matrix metalloproteinase; P, post-natal day.

The lung is the key organ of gas exchange in air-breathing mammals. This gas exchange structure is derived from the primitive foregut and proceeds through a phase of early (embryonic) development (1–3), when the conducting airways and conducting vessels are generated and organized (4). Early lung development initiates with the embryonic stage that occurs 4–7 weeks post-conception in humans [embryonic day (E)9–E12 in the mouse]. The embryonic stage is followed by the pseudoglandular stage, which occurs at 5–17 weeks post-conception in humans (E12–E17 in mice). The final stage of *early lung development* is the canalicular stage, occurring at 16–26 weeks post-conception in humans (E17–E18 in mice), at which point, the process of alveolarization begins, which is characterized by the thinning of the interstitial tissue (Figure 1). This marks the beginning of *late lung development*, where the distal airways then form saccular units in the saccular stage, which is evident in humans at 24–38 weeks post-conception [E18-post-natal day (P)4 in mice], and these saccular units are divided by secondary septa (the process of “secondary septation”) during the alveolar stage, which is evident at 36 weeks post-conception to 36 months post-natal (and beyond) in humans (P4–P28 in mice). The objective of late lung development is the production of a large number of small alveoli, the principal gas exchange units of the lung. This process, which is poorly understood, creates a large surface area over which gas exchange takes place. Current knowledge on late lung development implicates transcription factors and epigenetic effects, which together regulate genetic programs driving lung development. These programs work in concert with contact- and growth factor-mediated cell–cell communication (5–7) to drive lung development. The development of the lung is also driven in part by physical forces from breathing motions and the production and remodeling of the extracellular matrix (ECM) scaffold.

Multiple diseases are complicated by disturbances to lung development. Notable among these is bronchopulmonary dysplasia (BPD), which affects prematurely born infants with acute respiratory failure that receive oxygen therapy, first described by William (Bill) Northway and colleagues in 1967 (10, 11). While oxygen supplementation is a life-saving intervention, the associated oxygen toxicity stunts the post-natal development of the lung. This damage to the developing lung is exacerbated by barotrauma and volutrauma caused by positive-pressure mechanical ventilation, and also by inflammation. Affected infants exhibit blunted lung maturation, and BPD represents a significant cause of morbidity and mortality in a neonatal intensive care setting (11–14). Longitudinal studies suggest that disease sequelae persist into adult life (15–17). Examinations of autopsy material from patients that have died with BPD have formed the basis of hypotheses about pathogenic processes at play that limit alveolarization. These observations include (i) severe disturbances to the development of the pulmonary vasculature (18), (ii) changes in the cellular structure and composition of the developing alveolar units, (iii) increased proteolysis in the alveolar compartments, (iv) increased inflammatory cell infiltration, (v) deregulated growth factor signaling, and (vi) perturbations to the ECM architecture of the developing lung: most notably, the abundance and organization of collagen and elastin fibers (19–22). These disturbances have also been noted in animal models of BPD (23, 24).

It is the objective of this review to highlight key observations made regarding changes to the ECM architecture of the lung – both in clinical BPD and in experimental animal models of BPD (referred to herein as “experimental BPD”) – and to integrate these observations into a pathogenic pathway. Furthermore, attention will be paid to current controversies in the field, and also, to the key gaps in our knowledge, where urgent additional work is still to be undertaken.

## Early Studies: The ECM in Lung Development and BPD

The ECM represents a very complex network of structurally, mechanically, and biochemically heterogeneous components (25). The components include the classic “players”: collagen and elastin, which constitute 50% (26) and 18% (27), respectively, of the lung ECM. This list continues to grow, with fibrillin (28) and fibulin (29) glycoproteins, and integrin receptors of ECM components (30) being more recent additions. The ECM serves as a scaffold that directs lung development, and the ECM structure itself is continuously remodeled as lung development proceeds (31, 32). As such, the production of ECM components, as well as the systems that regulate the deposition and stability of the ECM, must be considered. These systems include chaperones and enzymes that catalyze the post-translational processing of ECM components, as well as systems that destabilize and degrade the ECM to facilitate ECM renewal. The remodeling of collagen during lung development has been recognized since the early 1970s (33). Pioneering work by Ron Crystal’s group identified the heterogeneity of fibrillar collagens in the lung during lung development (34–36). Observations on the dynamic remodeling of other collagen types, including basement membrane-type IV collagen then followed, in the context of early lung development (37, 38). Similarly, pioneering work by Janet Powell and Philip Whitney in the early 1980s described changes in lung elastin levels as post-natal lung development proceeded (39). Subsequent work by Ron Crystal (40) and Robert Rucker’s (41) teams highlighted the dynamic expression of tropoelastin during the course of post-natal lung development. Building on these studies, early work demonstrating that lathyrogens could disturb normal lung development (42) highlighted the role of collagen and elastin in the post-natal maturation of the lung. Soon to follow these reports were key observations of perturbed ECM structures in disorders of lung development. Leading work by Donald Thibeault and William Truog, in particular, identified secondary collagen fibers in the developing parenchyma of neonates with BPD that were “disorganized, tortuous, and thickened” (20, 21). Similarly, elastin fibers exhibited an abnormal structure in infants with BPD, both in the parenchyma (43–45) and in the vasculature (46). These early studies firmly established a role for proper lung ECM homeostasis in normal lung development, and described severe structural perturbations to the lung ECM that accompanied aberrant lung development. Clearly, it is important to note that it is sometimes difficult to establish whether the perturbations to ECM structure noted in clinical subjects with BPD are a cause of aberrant alveolarization, or a consequence of blunted lung development. This applies equally to pathological material from animals in which BPD has been modeled. Since these initial studies reported above, many strides have been made in our understanding of the production of the structural components of the ECM during post-natal lung development, which will be considered in detail below.

## Key Structural Components of the ECM: Collagen and Elastin

### Collagen

Collagen is the most abundant protein within the interstitial ECM. In the lung, collagen fibers [represented predominantly by the fibrillar collagens, collagen type I and III produced by fibroblasts (Table 1)] are found in the bronchi, blood vessels, and the alveolar septa (35, 47, 48). The abundance of lung parenchymal collagen increases over the course of lung development. In mice, gene expression of fibrillar collagens *Col1a1* and *Col3a1*, as well as basement membrane collagens *Col4a1* and *Col4a2*, is reported to have peaked at P7 (Figure 1). By this time, the collagen had formed a delicate interstitial network of fibers that could aid the process of alveolar development (8). While *Col1a1*^−/−^ mice, which lack collagen Iα_1_, died *in utero* due to the rupture of major blood vessels, no abnormalities were noted in lung branching morphogenesis in these mice (49, 50). However, elevated levels of other fibrillar collagens, including collagen III and V levels, were noted in *Col1a1*^−/−^ mouse embryos, suggesting a possible compensation for the loss of functional collagen I (50).

**Table 1:** **Cellular localization and origin of individual components of extracellular matrix and extracellular matrix remodeling enzymes**.

Extracellular matrix component	Origin/source				
	Epithelial cells	Fibroblasts	Endothelial cells	Smooth muscle cells	Inflammatory cells
Collagen		(51–53)			
EC-SOD	(54, 55)				
Elastin		(51, 53, 56)		(51, 57)	
Fibrillin-1		(58, 59)			
Fibronectin		(58, 60–62)	(60)	(60)	(60, 61)
Fibulin-5/DANCE		(63)		(64)	
Heparan sulfate	(65)	(66)			
Integrins	(67–71)	(69)	(70)	(70)	
LOX		(72, 73)		(72, 73)	
LOXL1		(72, 73)		(72, 73)	
LOXL2		(72, 73)		(72, 73)	
LTBP2		(58)			
MMP-1	(74)	(74)			
MMP-14/MT1-MMP	(74–76)	(75)	(76)		
MMP-2	(74, 75, 77, 78)	(75)	(74)		(77)
MMP-9	(74, 77–79)	(61)			(77, 79)
PLOD1	(80)	(80)	(80)	(80)	
PLOD2	(80)	(80)	(80)	(80)	
PLOD3	(80)	(80)	(80)	(80)	
Tenascin C	(81, 82)	(81–84)	(83, 84)	(83)	(81)
TGF-β	(78)	(61)			
TGM2/tTG	(85–87)	(52, 85, 86)	(85)	(85)	
TIMP-1	(75)	(75)			
TIMP-2	(74, 75)	(75)	(74)		

*Numbers in parentheses indicate the citations reporting the identification of the extracellular matrix components or remodeling enzymes in the indicated lung cell types. The citations are not all inclusive, and represent only a selection of reports*.

*EC-SOD, extracellular superoxide dismutase 3; LOX, lysyl oxidase; LoxL1, lysyl oxidase-like 1; LoxL2, lysyl oxidase-like 2; LTBP2, latent TGF-β-binding protein 2; MMP, matrix metalloproteinase; MT1, membrane-type 1; PLOD, procollagen-lysine, 2-oxoglutarate 5-dioxygenase; TGF-β, transforming growth factor-β; TGM2, transglutaminase 2; TIMP, tissue inhibitor of metalloproteinase; tTG, tissue transglutaminase*.

Alterations to the structure and integrity of collagen networks have been reported in several animal models of BPD and emphysema, which are diseases of the lung parenchyma that represent a failure of alveolar formation and the destruction of existing alveoli, respectively (21, 72, 88). Studies in various BPD animal models have revealed increased collagen production (Table 2), thickened collagen fibers, and increased rigidity of the lung (72, 88, 89) to be associated with experimental BPD. This is consistent with observations made in clinical subjects, where an increased number of collagen-positive cells, elevated levels of the fibrillar collagens, collagen I and collagen III were observed; and BPD patients revealed a specific increase in the collagen I/collagen III ratio (48, 90). Furthermore, elevated levels of collagen IV (91) have been noted in bronchoalveolar lavage (BAL) fluids from patients with BPD. These observations are supported by microscopic studies on patient tissues. Thibeault and colleagues (21) observed thickened and disorganized collagen fibers, and a generally damaged collagen network in the lungs of infants diagnosed with BPD after positive-pressure ventilation. It was proposed by those investigators that enlargement of alveoli due to ventilation leads to compression of surrounding ECM structures and damage to the collagen and elastin niche, disturbing the normal septation process. However, both adult rats (92) and newborn mice (93) exposed to sub-lethal normobaric hyperoxia up-regulated collagen I production, assessed by northern blot and immunoblot, respectively. In the case of newborn mice, the increased collagen I production was attributed to activation of the pro-fibrotic growth factor, transforming growth factor (TGF)-β, which stimulated collagen production and secretion by fibroblasts. Increased collagen deposition in the lung parenchyma of newborn mice has been confirmed in the hyperoxia-based mouse BPD model by picrosirius red staining (72, 94). Additionally, total lung collagen protein levels were increased by 63% after exposure of developing mouse pups to hyperoxia (89). Taken together, these reports make a strong case for dysregulated collagen expression in aberrant lung development associated with clinical and experimental BPD.

**Table 2:** **Dysregulation of the expression of extracellular matrix components and remodeling enzymes in clinical bronchopulmonary dysplasia and experimental animal models**.

ECM component	Expression in the disease/experimental condition		
	Bronchopulmonary dysplasia	Hyperoxia	Mechanical ventilation
Collagen	↑ (48, 91)	↓ (Fibroblasts, *in vitro*) (95)	
		↑ (Mouse) (72, 89, 93)	
		↑ (Rat) (92)	
EC-SOD		↓ (Mouse) (55)	
Elastin		↓ (Fibroblasts, *in vitro*) (95)	↑ (Mouse) (23, 99–101)
		↓ (Mouse) (89, 96)	↑ (Lamb) (24, 102)
		↑ (Mouse) (51, 72, 93, 97)	↑ (Rat) (103)
		↑ (Rat) (98)	
Fibrillin-1		↑ (Mouse) (51)	↑ (Mouse) (99)
			↑ (Lamb) (24)
EMILIN-1		↑ (Mouse) (23, 72)	↓ (Mouse) (23)
Fibrillin-2		↑ (Mouse) (51)	↓ (Mouse) (23, 99)
Fibronectin	↑ (60, 62, 104, 105)	↑ (Mouse) (105)	
		↑ (Rabbit) (106)	
Fibulin-5/DANCE		↑ (Mouse) (51, 72)	↓ (Mouse) (23)
			↑ (Rat) (103)
			↑ (Lamb) (24)
Integrins		↑ (Mouse) (51)	
Lox	↑ (72)	↑ (Mouse) (51, 72, 89)	↑ (Mouse) (23)
			↑ (Lamb) (24)
Loxl1	↑ (72)	↑ (Mouse) (72, 89)	↓ (Mouse) (23)
			↑ (Lamb) (24)
			↑ (Rat) (103)
Loxl2		↑ (Mouse) (72, 89)	
MMP-1		↑ (Rat) (92)	↓ (Baboon) (107)
MMP-16		↓ (Rat) (108)	
MMP-2	↓ (109)	↓ (Rat) (110)	
		↕ (Rat) (78)	
		↑ (Rat) (77)	
		↑ (Mice) (93)	
MMP-8	↑ (111, 112)		↓ (Baboon) (107)
MMP-9		↓ (Rat) (110)	↑ (Rat) (103)
		↕ (Rat) (78)	↑ (Mouse) (100, 101)
		↑ (Rat) (77)	↑ (Baboon) (107)
		↑ (Mice) (93)	
MMP-9:TIMP-1	↑ (113, 114)		↑ (Baboon) (107)
MT1-MMP		↑ (Rat) (78)	
PLOD1		↑ (Mouse) (80)	
PLOD2	↑ (80)	↑ (Mouse) (80)	
PLOD3		↑ (Mouse) (80)	
Tenascin C	↑ (83)	↓ (Fibroblasts, *in vitro*) (95)	↑ (Rat) (103)
TGF-β	↑ (115)	↑ (Mouse) (51)	↑ (Lamb) (24)
		↑ (Rat) (78, 92)	
TIMP-1	↓ (113)	↑ (Fibroblasts, *in vitro*) (95)	
		↑ (Rat) (78, 110)	
tTG	↑ (85)	↑ (Mouse) (85)	

*Arrows indicate the direction of dysregulated expression: ↓, down-regulation; ↑, up-regulation; ↕, temporal regulation in either direction over time*.

*ECM, extracellular matrix; EC-SOD, extracellular superoxide dismutase 3; EMILIN-1, elastin microfibril interfacer 1; LOX, lysyl oxidase; LoxL1, lysyl oxidase-like 1; LoxL2, lysyl oxidase-like 2; LTBP2, latent TGF-β-binding protein 2; MMP, matrix metalloproteinase; MT1, membrane-type 1; PLOD, procollagen-lysine, 2-oxoglutarate 5-dioxygenase; TGF-β, transforming growth factor-β; TGM2, transglutaminase 2; TIMP, tissue inhibitor of metalloproteinase; tTG, tissue transglutaminase*.

Collagen production under physiological and pathophysiological conditions is regulated by inter alia growth factors, such as TGF-β, where *in vitro* stimulation of primary lung fibroblasts drives *Col1a1* production (95, 116). This is significant, because elevated TGF**-**β levels were associated with BPD in preterm infants (115). TGF-β has also been causally implicated in the blunted alveolar development associated with hyperoxia exposure in the mouse hyperoxia model of BPD (117). The connection between TGF-β and collagen deposition in the developing lung is noteworthy. Over-expression of TGF-β driven by the *Scgb1a1* (encoding surfactant-associated protein C, pro-SPC) promoter in a doxycycline-inducible system is sometimes used as an animal model of BPD. Over-expression of TGF-β in this model not only resulted in blunted alveolarization but also increased deposition of collagen in the developing septa (118). Furthermore, over-expression of TGF-β in the developing lung *in utero* caused pulmonary hypoplasia that was accompanied by thickening of the collagen fibers and excessive collagen deposition in the septa (119). Exactly how the blunted alveolarization connects with perturbed ECM generation, both of which are guided by TGF-β, remains to be clarified.

Failed alveolar septation in both clinical and experimental BPD is clearly accompanied by changes to collagen production and deposition in the lungs. Studies, to date, have addressed primarily the fibrillar collagens collagen I and collagen III, however, the remaining 26 other collagens have received little or no attention. It remains of interest to explore whether perturbations to the expression of those collagens might be associated with arrested alveolar development. Similarly, no studies, to date, have examined the regulation or activity of the procollagen processing proteases, bone morphogenetic protein 1 (BMP-1) and ADAM metallopeptidase with thrombospondin type 1 motif, 2 (ADAMTS2). Both enzymes are required for procollagen processing and assembly into fibrils, during lung development.

### Elastin

Elastic fibers consist of extensively cross-linked elastin and fibrillin (28) microfibrils. These structures are associated with accessory molecules, including latent TGF-β-binding protein (LTBP), microfibril-associated proteins, fibulin, emilin, and microfibril-associated glycoprotein (MAGP) family members. Elastin fibers are located throughout the developing lung, in the developing conducting airways and alveolar ducts, the conducting vessels, and the developing septa. As illustrated in Figure 1, the expression of elastin in mice is dynamically regulated over the alveolarization period. Elastin expression dramatically increases at a time-point coincident with the “burst” of secondary septation that drives the formation of the alveoli. Elastin expression remains high throughout the secondary septation period [for example, in mice, over (P5–P15)] and rapidly decreases once alveolarization has been completed (8, 120). However, reactivation of elastin expression occurs in adult lungs under pathological conditions, such as emphysema and pulmonary fibrosis, where disorganized elastic fibers have been described (22, 120). The first hints that elastin plays a role in lung development included the observations that lung elastin levels were modulated as post-natal lung development proceeded (39). Additionally, the expression of tropoelastin, the “elastin monomer,” was dynamically regulated over the course of post-natal lung development in rodents (40, 41). During lung alveolarization, elastin is specifically deposited in “foci” at the tips of developing septa, suggesting a role in the process of secondary septation, which generates the alveoli. The spatially regulated deposition of elastin that coincides with secondary septation has led to the idea that elastin is a driver of lung development (121–123).

Further support for a role for elastin in lung development has been obtained using elastin-deficient mice. Elastin deficiency is accompanied by perinatal lethality, and *Eln*^−/−^ mice exhibit arrested perinatal development of the terminal airway branches, and enlarged terminal air sacs (124). Elastin haploinsufficient (*Eln*^+/−^) mice, which express 50% of the elastin seen in wild-type mice (125), exhibited normal lung development and normal alveolar structures, although there is some evidence that the elastin deposition in *Eln*^−/−^ mice was abnormal (99). Modulating the dose of elastin to <50%, by expressing the human elastin gene in a transgenic homozygous-null *Eln*^−/−^ mouse strain reduced elastin levels to 37% of wild-type mouse levels. While transgenic expression of human elastin rescued the perinatal lethality observed in *Eln*^−/−^ mice, a pronounced blunting of alveolar development was noted (125). These data indicate that a baseline threshold of elastin abundance is required for normal lung development to proceed. All of these observations underscore important roles for the correct spatio-temporal production of elastin structures in the developing lung.

In the context of lung disease, abnormal elastin fiber structures have been observed in the parenchyma of aberrantly developing lungs from prematurely born ventilated neonates (126). Parallel trends have been observed in animal models of BPD, where in response to mechanical ventilation or perinatal exposure to hyperoxia, the normally organized deposition of elastin fibers into foci at the tips of developing septa is lost. Rather, elastin fibers are noted in the walls (not the tips) of the thickened developing septa and have been described to be “brush-like,” “thickened,” and “loose” (32, 102, 127–129).

The pathological mechanisms behind the disturbed production and deposition of elastin in aberrantly developing lungs remains to be clarified, however, much work in this area has been already done, and remains ongoing. There is a body of evidence that suggests that expression of the *Eln* gene is up-regulated by hyperoxia in animal models of BPD, as revealed by real-time reverse transcription (RT)-polymerase chain reaction (PCR) analysis of mRNA pools from lung homogenates (51, 72, 97). The cell types reported to produce elastin in the lung are listed in Table 1, which include fibroblasts and smooth muscle cells. How hyperoxia modulates *Eln* gene expression might be attributed to growth factor stimulation or inhibition of elastin synthesis. Both TGF-β (130, 131) and insulin-like growth factor (IGF) (132) stimulated *Eln* gene expression, whereas some forms of platelet-derived growth factor (PDGF) suppressed *Eln* gene expression (133). Furthermore, the stability of *Eln* mRNA was increased by TGF-β, without impacting mRNA synthesis by lung fibroblasts (134). This is important, since increased TGF-β signaling and levels of TGF-β ligands were associated with experimental (117) and clinical BPD (115). Apart from TGF-β, increased IGF levels were also associated with experimental (135) and clinical (136) BPD, whereas decreased levels of some forms of PDGF were associated with clinical BPD (137). Taken together, these data would suggest that the pro-elastogenic effects of TGF-β and IGF were promoted, while the anti-elastogenic activity of PDGF was blocked during arrested alveolarization associated with BPD. These effects may also explain the increased abundance of *Eln* mRNA in the lung in hyperoxia-based experimental animal models of BPD.

It might be argued that given the extraordinarily long half-life of elastin fibers in the lung [estimated to be several years in the mouse (138)], studies on gene expression are less meaningful than studies on elastin protein production and organization into elastic fibers. Experimental studies on alveolarization tend to examine elastin distribution by light microscopy [for example, with Hart’s stain (72, 100, 101) or immunohistochemistry (51)], and infer elastin abundance from those studies. However, some studies have directly addressed insoluble elastin fiber abundance biochemically, where, in contrast to elevated mRNA levels, there appeared to be a paucity of insoluble elastin in affected lungs, assessed by lung desmosine or isodesmosine amounts (89, 96). The paucity of elastin was generally accompanied by the clearly disorganized structure and distribution of elastin fibers evident in the developing septa. This discord between elastin gene expression (which was increased) and the abundance of insoluble elastin (which was decreased) in injured developing lungs (together with perturbed elastin fiber structure and distribution) has several possible explanations, none of which have yet been experimentally tested. (i) The post-transcriptional regulation of *Eln* gene expression may be affected. For example, translation of mature *Eln* mRNA may be blocked by microRNA species generated in response to hyperoxia. Among the microRNA species that have been identified the target elastin are miR-29a/b/c (139) and miR-184, miR-194, miR-299, and miR-376b (http://www.mirbase.org). The possibility of microRNA regulation of elastin expression in the lung has not yet been addressed. Alternatively, the paucity of insoluble elastin in the background of increased *Eln* mRNA abundance might be attributed to (ii) defective post-translational maturation of elastin during fiber formation, or (iii) increased proteolytic degradation of elastin. Concerning post-translational maturation of elastin fibers, many accessory proteins have been identified that can associate with elastin fibers. These include the glycoproteins emilin (140), fibulin (29), LTBP (141), and MAGP family members (142). Discordant expression of these elastin fiber-associated proteins may result in unstable or malformed fiber structures. Indeed, Richard Bland has proposed that the uncoupling of elastin synthesis and assembly is a pathogenic contributor to disordered elastin fiber generation in BPD (23). Elastin fibers with abnormal physical properties may also result from the aberrant activity of the elastin maturation machinery, including the hydroxylation and cross-linking activities of lysyl hydroxylases and lysyl oxidases, respectively. These possibilities are discussed below. Alternatively, changes in the proteolytic capacity of injured, developing lungs may impact elastin fiber production or turnover, either directly (by proteolysis) or indirectly (by regulating the activity of mediators of elastin production). It is these lines of enquiry that are likely to further our understanding of *why* elastin organization is disturbed, and *what* impact this has on alveolarization in the developing lungs.

Some reports addressing the role of serine proteinases in the regulation of elastin production have already yielded exciting data. The group of Richard Bland has examined the utility of blocking serine peptidase activity in the context of BPD. Serine peptidase activity, such as that of neutrophil elastase, was elevated in the lung in clinical and experimental BPD. Mechanical ventilation of mouse pups with 40% O_2_ increased elastin degradation and disturbed septal elastin fiber deposition in the mouse lung, which was prevented by intratracheal administration of the neutrophil elastase inhibitor elafin (100). Thus, inhibition of neutrophil elastase activity [and probably matrix metalloproteinase (MMP)-9 activity as well, since MMP-9 can also be inhibited by elafin] partially restored proper elastin structures and improved lung alveolarization in this model. Furthermore, inhibition of neutrophil elastase activity blunted inflammation and inhibited the generation of active TGF-β that was proposed to be released from the ECM by proteolysis. In support of this idea, transgenic over-expression of elafin in the vascular endothelium similarly protected mice against the aberrant alveolarization and perturbed elastin assembly caused by mechanical ventilation (101). Subsequent exciting work by Keith Tanswell’s group has similarly reported that neutrophil elastase inhibition with sivelestat also improved lung structure and elastin deposition in the hyperoxia-based BPD animal model in mice (98). In this study, it is also noted that administration of anti-elastin antibodies in the mouse hyperoxia model of BPD prevented inflammatory infiltration into the lungs. Thus, these investigators raised the exciting possibility that neutrophil elastase-generated elastin fragments acted as pro-inflammatory matrikines (143), suggesting a mechanism by which hyperoxia exposure provoked lung inflammation. These data also raise further questions, for example, while neutrophil elastase inhibition clearly improved alveolarization in two different animal models of BPD, the underlying mechanisms remain unclear. The organization of elastin fibers was improved in both models, and inflammation and TGF-β activation was blunted. However, it remains unclear whether the improved alveolarization was a direct or indirect consequence of elastase inhibition (144). For example, was the generation of elastin fragments sufficient to provoke lung inflammation, or did the elastase-mediated activation of TGF-β play a role in this process as well? Elastase inhibition in the background of TGF-β neutralization would go some distance to resolving these open questions.

One vexing controversy in the lung alveolarization field is: are elastin protein levels elevated or reduced in the aberrantly developing lungs in the hyperoxia-based animal models of BPD? In mechanically ventilated lambs and mice, multiple reports document increased *Eln* mRNA levels, which were consistent with increased elastin protein levels in the lung (23, 102). However, this was not the case with normobaric hyperoxia-based models in mice, where many reports also confirm that Eln mRNA levels were up-regulated by hyperoxia exposure, but there appeared to be a paucity of lung insoluble elastin, when (iso)desmosine was used as a surrogate for mature, insoluble elastin fibers (89, 96). However, these observations are complicated by other reports of *increased* elastin protein in the hyperoxia models, employing either slot–blots (98) or immunoblots (51, 93). This controversy must still be resolved. These discordant data might be attributable to the methodology employed, where protein extraction by sodium dodecyl sulfate (SDS)-polyacrylamide gel electrophoresis (PAGE) for the blot-based protocols may have a different capacity for the extraction of insoluble elastin compared with the whole-lung hydrolyzates used in the (iso)desmosine approaches. Irrespectively, neither approach address the quantification of elastin specifically in the developing septa, which represents a major limitation of all of the approaches currently employed.

The current state of the field seems to suggest less lung elastin and more lung collagen, at least in the hyperoxia models of BPD. Given that, collagen imparts rigidity and elastin imparts elasticity to the lung, a shift in the collagen:elastin ratio may impact alveologenesis. This shift in collagen:elastin ratio may be as much as threefold increased by hyperoxia exposure (89). This is likely to dramatically impact lung compliance, and given the importance of the physical forces generated by breathing motions in “pulling the alveoli into shape,” a shift in the lung collagen:elastin ratio cannot be discounted as a possible contributing factor to lung alveolar development.

## Additional Structural Components of the ECM

### Fibrillins

**Figure 2:**
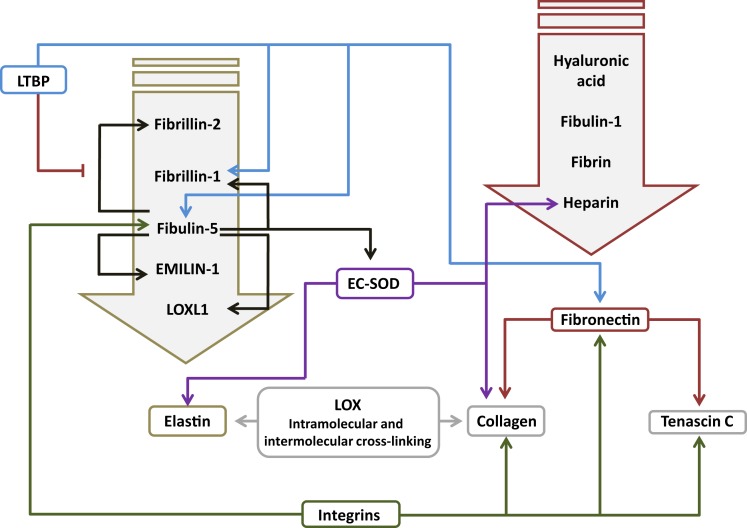
**Interactions between components of the extracellular matrix in the lung**. The primary interacting molecules for elastin and fibronectin are collected together above the respective target molecules in the downward-pointing block arrows. Abbreviations: EC-SOD, extracellular superoxide dismutase; EMILIN-1, elastin microfibril interfacer 1; LOX, lysyl oxidase; LOXL1, lysyl oxidase-like 1; LTBP, latent transforming growth factor-β-binding protein.

Fibrillins are elastin-binding glycoproteins (Figure 2) that make up the bulk of the microfibril component of elastic fibers, and act as a scaffold for elastic fiber deposition (28). Fibrillin-1 (Fbn1) and fibrillin-2 (Fbn2) are the main microfibril proteins (145). Fbn1 is clearly important for alveolarization and the structural homeostasis of the alveoli, since *Fbn1*^−/−^ mice exhibited an alveolarization defect (146), and fibrillin fibers were fragmented and disorganized in emphysema (147). In addition to imparting structural properties to elastic fibers, fibrillins may also help to mediate elastic fiber assembly, such as lysyl oxidase cross-linking of elastin fibers (28), which is thought to be highly relevant to lung development (27, 64, 72, 89, 148, 149). Similarly, Fbn1 played a role in anchoring LTBP to ECM components (58). Changes in fibrillin expression have been noted in animal models of BPD. In mechanically ventilated mice, the ratio of Fbn1:Fbn2 was increased, with elevated Fbn1 expression and reduced Fbn2 expression noted (23, 99). By contrast, in the mouse hyperoxia model of BPD, the expression of both *Fbn1* and *Fbn2* mRNA was elevated (51). With these ideas in mind, disturbances to fibrillin expression may impact lung development either by directly modulating the physical properties of elastic fibers or by altering TGF-β dynamics in the ECM. These ideas await experimental investigation.

### Tenascin C

Tenascins are a five-member family of large ECM glycoproteins, with tenascin C (Tnc), which is expressed in myofibroblasts, and endothelial, smooth muscle, and type II cells (81, 83), being the most studied in lung development (84) (Table 1). Tnc was comparatively highly expressed during human and animal development, including in the lung, particularly during the pseudoglandular and canalicular stages (84), at sites of active branching. Tnc is important for alveolarization, since *Tnc*^−/−^ mice exhibited an alveolarization defect (150). Along these lines, administration of dexamethasone to developing mouse pups blunted alveolarization, which was accompanied by decreased Tnc expression (56), although the impact of dexamethasone on Tnc expression was not causally linked to the blunted alveolarization. In contrast to these findings, *TNC* expression was elevated in the lungs of patients with BPD (83), which is consistent with the ability of TGF-β to drive *Tnc* expression in primary mouse fibroblasts *in vitro* (95). Tnc clearly plays a role in normal lung alveolarization; however, a causal role for changes on Tnc expression in aberrant lung alveolarization has yet to be demonstrated.

### Fibronectin

Fibronectin (Fn1) is a large (440 kDa) glycoprotein dimer, consisting of two almost identical subunits. Fn1 has been reported both as a soluble form in plasma and as an insoluble form associated with the ECM, where Fn1 binds collagen (Figure 2), as well as Tnc, and other ECM components (151). Fn1 is expressed in the lung (152), in interstitial fibroblasts, endothelial cells, and smooth muscle cells, but not in epithelial cells (Table 1). Fn1 expression was highest during lung development, and very low in adult lung tissue (152). *Fn1*^−/−^ mice exhibited early embryonic lethality (153), and a role for *Fn1* in lung development has not been demonstrated but is assumed. Several studies have documented the increased expression of Fn1 in clinical BPD, including in plasma, in endotracheal aspirates, and in BAL fluid (60, 104, 154, 155), as well as in lung tissue (60). This is consistent with the ability of TGF-β to drive Fb1 expression in lung fibroblasts (152). To date, no causal role for Fb1 in normal or aberrant lung development has been demonstrated. However, one exciting observation has suggested that decreased miR-206 expression in both clinical and experimental BPD may underlie the increased levels of *FB1* noted in the lungs of BPD patients (60, 105), since *FB1* has been described to be a target of miR-206 (105). Furthermore, miR-206 levels were decreased, whereas *Fb1* levels were increased in lungs from hyperoxia-exposed mouse pups (105). Taken together, these data make a compelling argument for the miR-206/Fb1 axis in aberrant alveolarization associated with BPD, although this idea requires experimental demonstration.

### Fibulins and Emilins

Fibulins and emilins promote proper elastin fiber formation, by mediating protein–protein interactions between ECM proteins, or between the ECM and ECM remodeling enzymes, such as lysyl oxidases (156). Fibulins are small calcium-dependent glycoproteins that bind elastin (Figure 2). Fibulin-5 (Fbln5; also called developmental arteries and neural crest EGF-like protein, DANCE) has been reported to play a role in lung alveolarization. *Fbln5*^−/−^ mice exhibited short, fragmented, and thickened elastin fibers, as well as a pronounced arrest of alveolarization (29, 157). No studies have examined a role for fibulins in clinical BPD; however, studies in animal models of BPD consistently revealed increased expression of Fbln5 in mouse pups exposed to hyperoxia (51, 72). Since TGF-β can drive Fbln5 expression (158), increased Fbln5 production after hyperoxia exposure may have been due to the attendant increased TGF-β signaling seen in this model (95). Changes in fibulin expression also appear to be sensitive to mechanical ventilation, where Martin Post’s group demonstrated that *Fbln5* expression was impacted by the duration and intensity of tidal volume ventilation, and breathing frequency, when rats were mechanically ventilated with room air (103). Conversely, Richard Bland’s group did not detect any impact of mechanical ventilation on Fbln5 expression when mice were ventilated with room air; however, ventilation with 40% O_2_ reduced Fbln5 levels in the lung, which was accompanied by blunted alveolarization. While the *Fbln5*^−/−^ mouse studies have implied a role for Fbln5 in alveolarization, the impact of increased Fbln5 expression on secondary septation and the development of the alveoli await demonstration. By way of speculation, Fbln5 promoted activation of MMP-2 and MMP-9 (159), which have also been associated with clinical and experimental BPD (see below). This, together with the possibility that Fbln5 over-expression might disturb elastic fiber formation, and might regulate the association of superoxide dismutase (SOD) (160) and lysyl oxidase-like 1 (LoxL1) (64) with the ECM, suggests avenues by which fibulin over-expression may influence lung development.

Like fibulins, emilins are a related group of elastic fiber-associated proteins (Figure 2), which impact elastogenesis, and have been reported to be expressed in the lung (161). Elastin microfibril interfacer 1 (Emilin-1) expression has not been studied in clinical BPD. However, Emilin1 expression has been reported to be dysregulated in animal models of BPD, including the hyperoxia exposure [*Emilin1* mRNA expression up-regulated; (23, 72)] and mechanical ventilation [Emilin1 protein expression down-regulated; (23)] in mice. *Emilin1*^−/−^ knockout mice do exist (162), although no lung phenotype has been reported. However, the reported dramatic (2,000-fold) up-regulation of *Emilin1* expression in c-Jun N-terminal kinase (Jnk) knockout mice was reported to be accompanied by an alveolarization defect, perhaps implicating Emilin1 in the alveolarization process (163), although a dramatic up-regulation of *Fbln1*, *Fbln5*, and *Eln* expression was also noted in that study.

### Latent TGF-β-Binding Proteins

The LTBP family consists of four extracellular MAGPs (164), which interact with, thereby modulate the activity of TGF-β. Ltbp1, Ltbp3, and Ltbp4 are reported to all associate with the small latent complex of TGF-β ligands and latency-associated propeptide (LAP), to generate the large latent complex (164). The LTBP family members are structurally related to fibrillins and were reported to interact with the ECM and play a role in ECM assembly. *Ltbp1*^−/−^ mice exhibited perinatal lethality with heart defects, while a lung phenotype was not studied or reported (165). By contrast, both *Ltbp3*^−/−^ and *Ltbp4*^−/−^ mice exhibited an arrest of alveolarization (166) that was more pronounced in *Ltbp4*^−/−^ mice (167). Ltbp4, which is known to bind Fbln5 (167), is believed to independently modulate elastogenesis and TGF-β activity, and thus, regulate lung development (168). The function of Ltbp2 remains elusive (164), but it has been suggested that Ltbp2 plays a TGF-β-independent role in elastogenesis (141), and Ltbp2 has been co-localized with fibronectin and Fbn1 in lung fibroblast cultures (58). Studies on Ltbp2 are complicated by the embryonic lethality reported in *Ltbp2*^−/−^ mice (169). Interestingly, despite a clear role in alveolarization, no studies, to date, have examined the expression of LTBP family members in clinical or experimental BPD. These exciting studies await experimental investigation.

### Polysaccharide Conjugates

Heparin, heparan sulfate, hyaluronic acid (hyaluronan), and chondroitin sulfate are polysaccharides or polysaccharide conjugates that have been reported to be mediators of lung alveolarization (170–172). Proteins carrying these conjugates, such as syndecan, which contains both heparan sulfate and chondroitin sulfate, exhibited molecular polymorphism – notably changes in the length of the heparin sulfate chains – over the course of lung development (173), implicating a role for heparan sulfate proteoglycans in lung development.

Temporal and spatial changes in glycosaminoglycan synthesis by lung fibroblasts have also been reported during lung development (174). Notably, fibroblasts in close proximity to the epithelium secreted hyaluronan, while more distant fibroblasts produced heparan sulfate and chondroitin sulfate during the pseudoglandular stage of lung development. During later stages of lung development, these fibroblasts switched to producing more hyaluronan, which was coincident with the thinning of the alveolar walls during the canalicular and later developmental stages. These authors postulated that developmentally regulated glycosaminoglycan generation by lung fibroblasts facilitated lung epithelial–mesenchymal interactions, which guided aspects of lung development (174).

Heparin and heparan sulfate have been reported to be the predominant glycosaminoglycans in epithelial basement membranes of the alveolus, and granules associated with collagen fibers of the basement membrane contained proteoglycan aggregates, which included chondroitin or dermatan sulfate (175). Heparan sulfate has been localized in the basement membrane during the embryonic, canalicular, and later phases of lung development (176). Heparan sulfate has received particular attention as a growth factor-binding protein, particularly in the context of fibroblast growth factor (FGF)-10, where Wellington Cardoso’s group has provided evidence that FGF-10 induction of local budding during early lung development is directed by developmentally regulated regional patterns of heparan sulfate sulfation (177). This idea has also been extended to cytokines, such as interleukin (IL)-1 in the developing chick lung (178), as well as members of the bone morphogenetic protein (BMP) family (179).

Several studies in transgenic mice have highlighted causal roles for enzymes of the heparan sulfate biosynthetic pathway in lung development. For example, deletion of *N*-deacetylase/*N*-sulfotransferase (heparan glucosaminyl) 1 (Ndst1) led to pulmonary hyperplasia and acute respiratory distress in mice, possibly due to decreased surfactant production as a result of type II cells to mature (180). Similarly, deletion of glucuronyl C5-epimerase (Glce) in mice caused embryonic lethality, and stunted embryonic lung development, which was accompanied by a total loss of l-iduronic acid in heparan sulfate conjugates (181). Specifically concerning late lung development, defects in the development of the airspaces have been noted in both heparan sulfate 6-*O*-sulfotransferase 1 (Hs6st1) (182) and sulfatase 2 (Sulf2) (183) knockout mice. Apart from mice lacking enzymes involved in the heparan sulfate biosynthetic pathway, mice lacking heparan sulfate proteoglycans also exhibit lung development phenotypes. For example, deletion of glypican-3, a member of a family of heparan sulfate proteoglycans linked to the cell surface through a glycosyl-phosphatidylinositol anchor, generated abnormal lung structures in mice (184). These studies validated the earlier suggestion that heparin and heparan sulfate are mediators of lung development, although most work has been confined to the earlier stages of lung development that precede alveolarization. The generation of antibodies that detect specific heparan sulfate epitopes has facilitated the identification of spatio-temporal changes in heparan sulfate structure during normal lung development, and aberrant lung development associated with congenital diaphragmatic hernia (CDH) (65, 185), which will facilitate further mechanistic work in this area. To date, exactly how these structural abnormalities to heparan sulfate proteoglycans results in disturbed alveolar structure remains to be clarified.

In addition to heparan sulfate proteoglycans, some work in embryonic lung explants has also revealed a role for chondroitin sulfate proteoglycans in early lung development (186). Furthermore, an interesting connection with inflammatory cells has been made, with the suggestion that CD44-positive macrophages, which take up hyaluronan, may regulate the steady-state levels of hyaluronan during lung development (187). Further work in this area should examine *how* defined alterations to proteoglycan structures direct proper development of the lung. The existence of transgenic mice and monoclonal antibodies that allow the specific detection of proteoglycan structures will facilitate these efforts. Additionally, no studies, to date, have examined changes in the expression of proteoglycan biosynthetic enzymes, or proteoglycan structures, in animal models of BPD.

## ECM-Interacting Molecules

### Integrins

Integrins are large heterodimeric transmembrane glycoproteins associated with various elements of the ECM. Integrin ligands include collagen I, Tnc, Fb1, laminins, TGF-β, and tissue transglutaminase (Tgm2), among many others. Each integrin dimer consists of a single α and β subunit. There are many integrin subunits, with 18 α and 8 β subunits having been identified in humans, to date (67). Expression of integrins is known to be dynamically regulated during lung development, where integrin-mediated cell–ECM interactions are known to play an important role (68, 188). Integrin expression has been noted during alveolarization, with the α2, α3, α6, and β1 subunits having been reported to be expressed in the bronchial and alveolar epithelium during the alveolar stage of lung development, as well as in adult lungs. By contrast, the α4 subunit was reported to be expressed in the respiratory epithelium only during lung development and has not been detected in adult lungs (68, 69, 188). The fibronectin receptor, integrin α8β1 (189) has been demonstrated to play a particularly noteworthy role in early and late lung development, where the Lawrence Prince’s group demonstrated that *in utero* exposure of developing embryos to bacterial lipopolysaccharide (LPS) caused a reduction in expression of *Itga8*, which encodes the α8 integrin subunit, in mesenchymal cells (30). Thus, these authors examined lung structure in *Itga8*^−/−^ mice, which exhibited a pronounced disturbance to the developing lung structure, including lobar fusion and alveolar simplification. Additionally, elastin fibers in these mouse lungs were described to be “wavy and short.” This led these authors to suggest that integrin–ECM interactions played a notable role in late lung development. This idea is supported by observations made in the mouse hyperoxia BPD model, where increased expression of *Itgav*, encoding the α_v_ integrin subunit [which also binds fibronectin; (67)], was noted (51), and was accompanied by impaired alveolarization and increased *Fbln5* expression and TGF-β activity, and aberrant elastin fiber deposition. These studies have opened up an exciting new avenue, that is, the role of integrin-mediated ECM interactions in the regulation of alveolarization.

### Extracellular Superoxide Dismutase

Extracellular superoxide dismutase (EC-SOD or Sod3), is one of three forms of SOD, a group of antioxidant enzymes representing the major cellular defense against the superoxide anion $\left({\text{O}}_{2}^{\cdot }\right)$ (54, 190). EC-SOD is the only ECM-related antioxidant and has been reported to be the most abundant SOD in the lung (191). EC-SOD was reported to be expressed primarily in vessels, large airways, and alveolar septa. EC-SOD binds heparin (192) and heparan sulfate proteoglycans on the cell surface, and components of the ECM (54). EC-SOD binds collagen I (Figure 2) and is thought to protect against oxidative damage to collagen I (193). EC-SOD is also known to bind to tropoelastin, a process that is mediated by Fbln5 (160). EC-SOD is believed to play a role in protecting the ECM from oxidative damage, since reactive oxygen species (ROS) drive elastin degradation and increased collagen cross-linking (194, 195). Thus, EC-SOD might protect the developing and adult lung from oxidative damage (54), since EC-SOD was reported to be expressed throughout life (196), although EC-SOD protein expression and activity were blunted by hyperoxia exposure in adult mice (197). Along these lines, adult *Sod*^−/−^ mice exhibited increased sensitivity to hyperoxic damage, with reduced survival and more pronounced alveolar edema, compared to wild-type mice; thus, supporting a role for EC-SOD in protection against oxidative damage to the lung (198). In support of this idea, over-expression of EC-SOD in transgenic neonatal mice protected against the damaging effects of hyperoxia on lung alveolarization (196), and expression of EC-SOD in a mouse lung epithelial cell-line protected against oxidative damage-induced cell death (55). The protective effects of EC-SOD over-expression on lung epithelial cells has also been demonstrated *in vivo* in hyperoxia-exposed newborn mice (199). None of these studies addressed collagen or elastin fiber integrity.

A wide spectrum of other ECM-interacting proteins still remains to be studied in the context of lung alveolarization. These proteins include the MAGP family members (142), as well as the small leucine-rich proteoglycans, such as decorin and related molecules, which play key roles in driving collagen fiber formation (200, 201). These future studies will no doubt add to the list of ECM-associated proteins that impact normal and aberrant late lung development.

## ECM Remodeling Enzymes

### Matrix Metalloproteinases and Their Inhibitors

**Figure 3:**
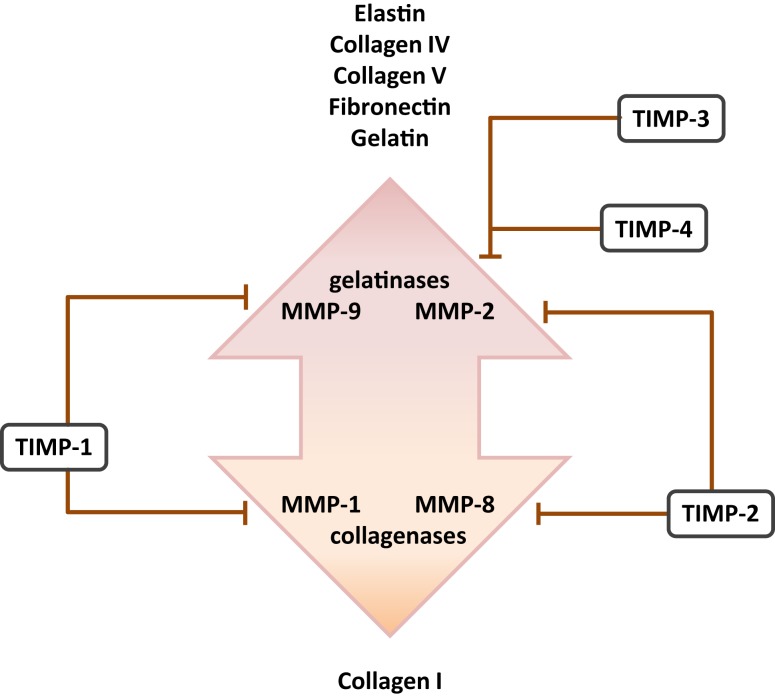
**Cognate inhibitors and target substrates of matrix metalloproteinases**. The target substrates of the gelatinase and collagenase members of the matrix metalloproteinase (MMP) family are indicated, together with selected target MMP substrates. Abbreviation: TIMPs, tissue inhibitor of matrix metalloproteinases.

Matrix metalloproteinases are a large family of endopeptidases responsible for ECM breakdown and remodeling, which are necessary processes for proper formation of the ECM (9, 202). Different MMPs preferentially degrade different components of the ECM, with MMP-1 and MMP-8 active against fibrillar collagens, and MMP-2 and MMP-9 preferentially active against basement membrane collagen (collagen IV), fibronectin, and elastin (77, 203–206). The proteolytic activity of MMPs can be regulated by MMP binding to cognate inhibitors, such as tissue inhibitor of metalloproteinases (TIMPs) (110, 207) (Figure 3). The expression of MMPs in the lung is known to be dynamically regulated over the course of lung development (Figure 1), with a progressive decrease in MMP-2 and MMP-14 [also called membrane-type-1 (MT1)-MMP] expression, but a progressive increase in MMP-9 expression between E10 and P21. These trends imply a role in lung alveolarization (9, 75, 78). Expression of MMP-2 and MMP-14 has been noted in airway and alveolar epithelial cells, endothelial cells, and fibroblasts (74–76), whereas MMP-9 was reported to be expressed in epithelial cells, fibroblasts, and inflammatory cells, including neutrophils and alveolar macrophages (61, 77, 79) (Table 1). MMP expression in the lung was driven by exposure of adult (77) and neonatal (93) rodents to hyperoxia. Similarly, elevated MMP expression has been noted in endotracheal aspirates or BAL fluid from preterm infants with BPD (109, 111–113). MMPs also played a role in alveolar destruction in experimental emphysema in mice (208). MMPs might impact alveolarization directly, through degradation of ECM components, or indirectly, through activation of growth factor pathways. For example, MMP-9 activated TGF-β signaling, which in turn stimulated lung fibroblasts to contract (61, 209). MMP-9 appeared to be able to influence lung alveolarization, since *Mmp9*^−/−^ mice exhibited worsened lung development, in a mouse model where lung alveolarization was blocked by over-expression of IL-1β (210). In an alternative hyperoxia-based BPD model, *Mmp9*^−/−^ mice were protected against the blunted alveolarization usually seen in the mouse hyperoxia BPD model (93). The reasons for these two discordant observations are currently unclear, however, may be related to the different models employed. Along the same lines, *Mmp14*^−/−^ mice initially exhibited a 40% decrease in alveolar surface area compared to wild-type mice early during post-natal lung development (76), which was accompanied by thickened elastin fibers. By contrast, *Mmp2*^−/−^ mice exhibited a “delayed” alveolarization, where an alveolarization defect was noted at P7, but alveolarization was normalized at P14 (76). It would be interesting to explore the impact of hyperoxia or mechanical ventilation of the *Mmp2*^−/−^ and *Mmp14*^−/−^ mice on alveolarization.

**Figure 4:**
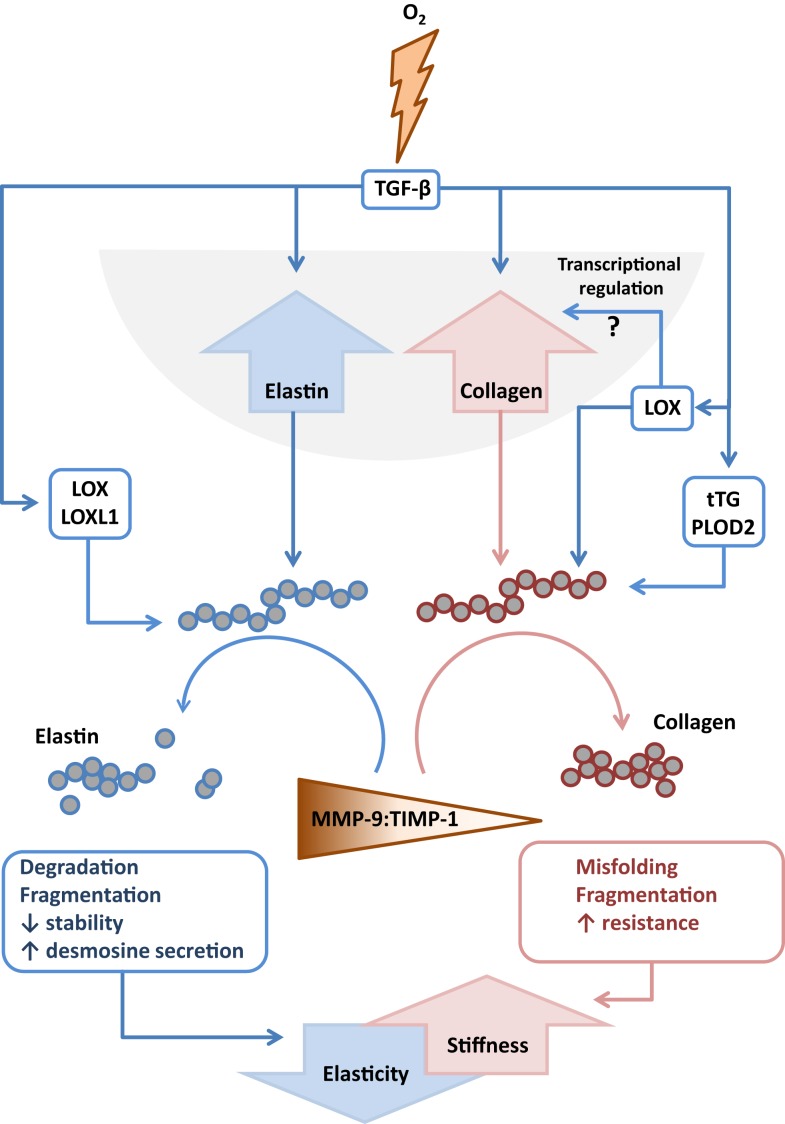
**Interactions between extracellular matrix components and remodeling enzymes driven by exposure to hyperoxia**. The extracellular matrix remodeling processes that are described to be driven by exposure of the developing lung to hyperoxia that lead to increased stiffness and decreased elasticity of the developing lungs. Abbreviations: LOX, lysyl oxidase; LOXL1, lysyl oxidase-like 1; MMP, matrix metalloproteinase; TGF-β, transforming growth factor-β; TIMPs, tissue inhibitor of matrix metalloproteinases; tTG, tissue transglutaminase.

Several studies have addressed MMP expression in clinical BPD cases, where reduced MMP-2 levels were noted in endotracheal aspirates (109) and plasma (211), but increased MMP-8 levels were noted in endotracheal aspirates (111) and BAL fluid (112) from preterm infants with BPD. Increased MMP-9:TIMP-1 ratios have also been detected in BAL fluids from preterm infants that developed BPD (114) (Figure 4). Additionally, Ekekezie and coworkers (113) observed an increased MMP-9:TIMP-1 ratio in endotracheal aspirates from BPD patients, which correlated with poor patient outcome. These trends largely parallel observations made in animal models of BPD, where increased levels of MMP-2 and MMP-9 proteins were noted in hyperoxia-exposed mouse pups (93). Similarly, MMP-9 levels were modulated in the lungs of hyperoxia-exposed rats (78), and increased MMP-9 levels and an increased MMP-9:TIMP-1 ratio were noted in a premature baboon BPD model (107). Not all trends in MMP expression are consistent between investigations. For example, Hosford and co-workers reported the *decreased* expression of MMP-9 and *increased* expression of TIMP-1 in the rat hyperoxia model of BPD (110), which was also accompanied by blunted alveolarization. These discordant observations might be attributed to the extraordinary variation in the application of the BPD models: (i) newborn rats exposed to >90% O_2_ for 9 days versus (ii) ventilated, premature baboons versus (iii) rats exposed to >95% O_2_ between P4 and P14. Irrespectively, the general trend is toward increased MMP-9 activity in aberrantly developing lungs.

### Lysyl Oxidases

Lysyl oxidases constitute a family of five members: the archetypical lysyl oxidase (Lox) and four lysyl oxidase-like enzymes (Loxl1–LoxL4) (212, 213). All lysyl oxidases catalyze the oxidative deamination of lysine and hydroxylysine residues, generating reactive semialdehydes, which then form intramolecular and intermolecular covalent cross-links in both elastin and collagen molecules (212, 213). Lysyl oxidases have been reported to play an essential role in normal lung development and have been implicated in the pathogenesis of several lung diseases, including pulmonary hypertension (73), lung adenocarcinoma (214), and BPD (72). Lysyl oxidases are known to play an important role in organogenesis, with *Lox*^−/−^ mice exhibiting perinatal lethality, ostensibly due to a failure of the cardio-respiratory system (215, 216). Furthermore, Lox has been specifically implicated in the development of the respiratory system, where Lox has been reported to be required for the integrity of elastic and collagen fibers in multiple tissues (27). Interestingly, genetic ablation exclusively of Lox expression significantly reduced total lysyl oxidase activity, suggesting that Lox is the primary contributor out of the five family members, to lysyl oxidase-mediated effects. In *Lox*^−/−^ mouse lungs, both desmosine and hydroxyproline levels were decreased relative to wild-type mice (216). While viable, *Loxl1*^−/−^ mice exhibited connective tissue weakness, and developed pelvic organ prolapse and *cutis laxa* (64). Furthermore, *Loxl1*^−/−^ mice exhibited alveolar simplification and reduced lung desmosine levels, implying a role in lung development, as well as perturbed elastin fiber structures throughout the organism (64).

Lysyl oxidase expression has been studied in animal models of BPD. Increased Lox and Loxl1 expression has been noted in the lungs of preterm ventilated lambs (24). Lox expression was also increased by mechanical ventilation in mouse pups (23), but lung Loxl1 levels were reduced in mechanically ventilated mice (23). Elevated lysyl oxidase activity (72, 89, 94), and elevated Lox and Loxl1 levels (72, 89) were detected in newborn mice exposed to normobaric hyperoxia. This has led some investigators to propose that the ECM in affected lungs might be “over cross-linked,” and thus excessively stabilized, which has been proposed to be a potential contributing factor in arrested alveologenesis associated with clinical and experimental BPD (72, 89). Consistent with elevated Lox expression and activity, exposure to hyperoxia also generated increased amounts of insoluble collagen and the dihydroxylysinonorleucine (DHLNL) collagen cross-link, as well as an increased DHLNL:hydroxylysinonorleucine (HLNL) ratio, and disordered elastin organization in the alveolar septa (89). To address a causal role for lysyl oxidases in blunted alveolarization in the hyperoxia BPD model, newborn mice were treated with the pan-lysyl oxidase inhibitor β-aminopropionitrile (BAPN), which did not improve lung alveolarization, but did improve elastin organization assessed by visual inspection (89). This might indicate that the partial normalization of elastin organization in developing septa alone (through normalization of lysyl oxidase activity) was not sufficient to normalize lung alveolarization in the mouse hyperoxia BPD model. Several questions regarding lysyl oxidases and alveolarization come to mind, among them: what role do the different lysyl oxidases play in lung development, and in which tissues? Lysyl oxidases are expressed in several different cell types (Table 1), and it may be that different lysyl oxidases have different contributions to lung alveolarization, acting in different cell types. The generation of conditional, inducible deletions of the various lysyl oxidase genes would help to address this question. Additionally, “non-matrix” roles for lysyl oxidases should also be considered, where lysyl oxidases have been reported to modulate gene regulation in the nucleus, for example, the expression of *COL3A1* (217). This has revealed nuclear functions – primarily of LoxL2 – which modulated epigenetic effects in the nucleus by deamination of trimethylated Lys^4^ in histone H3, which was linked to transcriptional repression (218). Furthermore, LoxL2 regulated keratinocyte differentiation independent of lysyl oxidase catalytic activity (219). Similar studies have yet to be performed with other lysyl oxidases, but highlight possible roles for lysyl oxidases in lung alveolarization that are not related to ECM cross-linking.

### Lysyl Hydroxylases

The ability of lysyl oxidases to generate covalent cross-links requires lysine or hydroxylysine residues in ECM substrates. These hydroxylysine residues are generated by another family of enzymes, the lysyl hydroxylases (officially named procollagen-lysine, 2-oxoglutarate 5-dioxygenases, or PLODs) (220), which consist of three family members: PLOD1–PLOD3. A role for lysyl hydroxylases in organ development was underscored by the early embryonic lethality of *Plod3*^−/−^ mice (221), while *Plod1*^−/−^ mice were viable, but exhibited vascular pathology and abnormal collagen fiber structure (222). This family of ECM-modifying enzymes is relatively poorly characterized. Some evidence does exist illustrating that lysyl hydroxylases play a role in aberrant late lung development, both in humans and in mice. A recent study by Witsch and colleagues (80) revealed that the lung expression of PLOD family member PLOD2 was up-regulated in premature infants with BPD. Furthermore, *Plod1*, *Plod2*, and *Plod3* expression was elevated in the lungs of mice in the hyperoxia BPD model (80), and the elevated *Plod2* expression was mediated by TGF-β. These data indicate that the lysyl hydroxylases may play a role in normal and abnormal lung development, and this possibility awaits experimental attention.

### Transglutaminases

The transglutaminases constitute an eight-member family of calcium-dependent enzymes, which cross-link collagens and fibronectin, among other proteins (223). Of the transglutaminases, largely transglutaminase 2 (Tgm2; also called tissue transglutaminase, tTG) has been studied in lung disease and was reported to be expressed in fibroblasts, as well as epithelial, endothelial, and smooth muscle cells (85, 224). In addition to cross-linking activity, Tgm2 is an integrin-binding adhesion co-receptor for fibronectin (225). Tgm2 has also been implicated in lung fibrosis (86, 226, 227), allergy (87), cystic fibrosis (228, 229), and pulmonary hypertension (230). Tgm2 has further been credited with a role in organogenesis (231), including lung development (232). In preterm infants with BPD, *TGM2* mRNA levels were elevated (85), which was also seen in the lungs of hyperoxia-exposed newborn mice with experimental BPD (85). In the case of hyperoxia-exposed newborn mice, increased *Tgm2* levels were driven by TGF-β, most likely in lung epithelial cells. This is particularly noteworthy because not only can TGF-β drive Tgm2 expression but Tgm2 can also activate TGF-β (233), suggesting a possible vicious circle of Tgm2 expression and TGF-β activation in aberrant lung alveolarization. These studies indicate that changes in transglutaminase expression are associated with normal and aberrant alveolarization; however, a causal role for transglutaminases in lung development has yet to be experimentally documented. The existing *Tmg2*^−/−^ knockout mice would be an ideal starting point for these studies (234).

## Perspective

Given that, the ECM plays a pivotal role in lung development, it comes as no surprise that perturbations to ECM production and remodeling accompany defective secondary septation and aberrant alveolarization associated with BPD. Identification of the perturbations to ECM organization that play a causal role in aberrant alveolarization would assist in our understanding of the pathological processes that disturb late lung development. Equally important is the delineation of pathogenic pathways that drive these causal disturbances to ECM structure.

Roles for ECM structural proteins and ECM remodeling enzymes in lung alveolarization have been identified using gene knockout approaches. These studies have provided a very solid foundation for future work but are complicated by the pre- or peri-natal lethal phenotype of some knockout mice. This has been partially remedied by the parallel over-expression of human genes, or genes with altered promoter activity, in the background of a homozygous-null strain (such as the expression of the human *ELN* gene in *Eln*^−/−^ mice, described above), which overcome the lethality of the homozygous-null mutants, and facilitated further studies on the gene products of interest. However, this has been more the exception than the norm. Additionally, it is widely recognized that the discrete expression of particular *genes*, in particular, *cell types* at particular *stages* of lung development is the basis of the highly coordinated program of the generation of a very complex organ (1, 2). This makes the use of constitutive global knockout mouse strains problematic.

Rapidly evolving mouse transgenic technology makes an increasing number of conditional-ready gene-deletion strains available through the use of floxed alleles. In combination with inducible Cre-recombinase systems, these conditional strains become inducible, conditional strains, which facilitate gene deletion in developing mouse pups at particular time points during post-natal lung development, in restricted cell types. These approaches rely largely on the use of doxycycline-inducible rtTA (*tetO*)_7_-Cre and tamoxifen-inducible Cre^ERT2^ systems. These inducible, conditional-ready mouse strains will prove invaluable in assessing how the temporal and tissue-specific expression of particular genes during lung development impacts lung development *per se* (235). Among the drawbacks of this approach are the limitations of some floxed allele strains, which would have to be created *de novo*, and also, the lack of – or technical difficulties with the use of – some driver lines. For example, no suitable driver line currently exists that can exclusively target lung fibroblasts, or that can discriminate between airway and vascular smooth muscle cells (235). Remaining with transgenic mice, most studies, to date, have evaluated the loss of a particular gene on lung development. However, particularly in the context of animal models of BPD, genes might be over-expressed or up-regulated, rather than down-regulated. As such, to be able to “phenocopy” a lung phenotype by over-expressing a gene of interest, *in the correct cell-type at the correct time*, would go a long way to validate candidate pathogenic mediators of arrested alveolarization. Along these lines, many knockout and pharmacological intervention studies have identified new “players” in normal lung alveolarization (such as LTBP and elastin and collagen cross-linking enzymes), but a contribution to pathological lung development in animal models of BPD has not been undertaken. These exciting studies may well reveal new pathogenic pathways that drive aberrant lung alveolarization.

While elastin has received a tremendous amount of attention as a regulator of lung development, the collagens remained largely neglected. Since many candidate pathogenic mediators (such as elastin cross-linking enzymes) also influence collagen structure and function, it would be interesting to explore roles for disturbed collagen organization during lung development. Along these lines, the ratio of elastin:collagen is also noteworthy. In the hyperoxia models of BPD, there is a reported shift toward an increased collagen:elastin ratio. This would impact lung rigidity and elasticity, and thus lung development, which is dependent on physical forces generated by, for example, breathing motions.

Animal models of BPD have proved very important for the identification of candidate pathogenic mediators of normal and aberrant late lung development. These studies are often not followed up with validation studies that pin-point a role (*if any*) for a particular candidate mediator that exhibited changes in gene or protein expression in a BPD model. These studies are important, since changes in the gene or protein expression of a particular molecule may be (i) epiphenomenal (i.e., that the molecule in question was a bystander without any role in the alveolarization process), (ii) causal (i.e., that molecule in question was a mediator of arrested alveolarization), or (iii) reparative (i.e., that molecule in question mediated a lung defense or repair program that was engaged during aberrant alveolarization, which aimed to restore proper alveolarization). It is very important to determine which of these three categories a “candidate” mediator of aberrant lung development falls into.

Interestingly, in many studies, once a candidate mediator of aberrant lung alveolarization was identified in an animal model of BPD, much effort was then expended on identifying how the candidate mediator impacted ECM structures during alveolarization. Rather, less energy is usually invested in understanding how the expression of the candidate mediator was altered by the injurious stimulus (for example, inflammation, hyperoxia, or mechanical ventilation). The identification of such proximal pathways would be important in a translational sense, where addressing the very proximal causes of arrested lung development might be therapeutically targeted, ultimately in affected patients. With this in mind, physical forces and oxidative stress might be good starting points to understand the activation of pathways that produce or remodel the ECM. Furthermore, ECM structures have recently been credited with a role in driving pluripotent cell differentiation in acellular lung scaffolds (236). Thus, how the ECM may shape stem cell niches in the developing lung, and direct phenotypic transformation of the constituent cell types of the developing lung are further areas that will no doubt receive attention in the coming years.

With the rapidly expanding repertoire of genetic tools, and the development of state-of-the-art methodology to study both lung alveolar architecture and the biochemical nature of the ECM, we have never been better positioned to explore the complex interactions of the ECM during lung alveolarization. It is clear that there is much exciting work to be done!

## Conflict of Interest Statement

The authors declare that the research was conducted in the absence of any commercial or financial relationships that could be construed as a potential conflict of interest.
